# Casualty Room Failures

**Published:** 1898-08-27

**Authors:** 


### CASUALTY ROOM FAILURES.

Mr. F. S. Langham held an inquest on Thursday, 18th inst., at Guy's Hospital concerning the death of Francis Naylor, aged ten years, the son of a clerk, living at 41, Howbury Road, Nunhead.

The mother of the deceased said that the preceding Friday the boy was playing in the garden with a younger brother. They had a sharp-pointed piece of wood, and the deceased fell over his brother. He screamed badly, and the witness on picking him up found the stick stuck in his eye. She took the child in a cab to Guy's Hospital.

The Coroner: Was he at once admitted?—No; they bandaged his eye, and I took him home, and brought him down again on Saturday.

Was he received into the hospital at once on Saturday?—No, sir; I brought him at a quarter-past one and I had to wait until half-past two before anyone saw him at all. They came and saw him then, and said he was very bad. He then went into a fit and did not recover, and died about twelve o'clock the next day.

Was he very much injured?—Yes; the stick was forced into his eye a good deal.

Did you tell that to the doctor?—Yes; I told the doctor when I brought him. I think I was kept a very long time at the hospital when they knew he was so bad.

Dr. Philip Turner, house surgeon, said he knew nothing whatever about the deceased until he was admitted to the ward under his charge, which was at about three o'clock. He was at once put to bed, and improved towards the evening, but died suddenly the next morning. A post-mortem examination revealed that the stick had penetrated nearly to the back of the orbit, and had caused a slight injury to the membrane covering the brain. This had set up meningitis, from which death ensued. Had witness seen deceased earlier he could not have done anything to save his life.

The Father: I make no complaint against this gentleman.

Dr. R. T. FitzHugh, assistant house surgeon, said he first saw the deceased on Saturday afternoon in the surgery, which was at that time crowded. Seeing the boy was perspiring freely and was very limp, which witness attributed to the hot weather and the sights about him, witness spoke to him but could get no reply. Witness gave him a stimulant and looked at his eye, and then the parents said he had been "seedy" during the night and morning. Witness said he would come back and look at him in a minute. Five or ten minutes later he was fetched to the boy, who was then in a fit, and he ordered his removal to the ward as soon as possible.

The Coroner: What about the waiting—the mother said she waited an hour and a quarter?—Yes, I daresay she did, sir. The only reason I can give is that the surgery was very busy at the time, and the boy had to see a Dr. Stoehr, that gentleman having an appointment with him. He, however, was no doubt busy elsewhere at the time. It was only by the fit that I recognised the gravity of the case, and therefore I think it was a good thing that the boy was kept waiting.

Why?—Because he might have been sent out.

The Father: If his eye had been examined when he first got to the hospital you would have seen how bad he was. You would not have seen him when you did if he had not been in a fit.—I beg your pardon; I looked at him.

The Father: There is great neglect somewhere.

A Juryman: I think the doctor ought not to have allowed him to go out of the hospital on Friday.

The Father: At any rate, when a doctor makes an appointment he should keep it, or get someone else to attend. Dr. Stoehr made an appointment with us for a quarter-past one, and we did not see him at all that day.

The Coroner: Where is Dr. Stoehr? We ought to have him here.

Dr. FitzHugh: He has gone for his holidays this afternoon.

The jury returned a verdict of Accidental death, and through the foreman expressed their opinion that Dr. Stoehr should have been present at that inquiry to explain why he did not keep his appointment on Saturday.

What the Guy's Authorities State.

We have received the following account of the case from Mr. Philip Turner (house surgeon at Guy's Hospital) and Mr. R. T. FitzHugh (the assistant house surgeon on duty the day after the accident):—

"The boy Naylor was brought to the hospital on Friday, August 12th, with the history of having run a stick while at play into his eye. He was seen by the dresser on duty, Mr. Stoehr, who called the attention of Mr. Booker, the assistant house surgeon then on duty, to him. A careful examination was made, and there was no evidence that serious injury had been sustained. His general condition was such that it was decided that he might safely be sent home, on the condition that he should be brought up the following day at quarter-past one to see Mr. Stoehr, a suitable dressing having been applied to the injured eye. At the appointed time his parents brought him up. Mr. Stoehr was then in a room leading out of the surgery dilating a stricture, and this naturally took him some considerable time. The assistant house surgeon on duty that day, Mr. FitzHugh, had casually noticed the boy, and he did not give the appearance of being urgently in need of attention. When Mr. Stoehr had finished with the stricture patient he came into the surgery, and at once went to the boy and brought him to the notice of Mr. FitzHugh. The father in his evidence stated that Mr. Stoehr never saw the boy at all on Saturday; this is incorrect, he evidently having forgotten Mr. Stoehr's appearance. Mr. FitzHugh, though busy, went at once to see the boy at Mr. Stoehr's request, and examined the eye; no evidence of any external wound was found, the ball was uninjured, but there was a good deal of bruising about the lids. Appropriate treatment was suggested, and Mr. FitzHugh was about to leave to look after other cases when Mr. Stoehr told him that the general condition of the boy had not been satisfactory during the night. A more complete examination was then made, when he was found to be sweating about the face, felt hot, was listless, would not answer questions, and had rather a feeble pulse. Not being sure how much of this was due to a passing faintness, it being a very hot day and the surgery crowded, Mr. FitzHugh ordered a dose of stimulant, and left while this was being got ready to attend to others, saying he would come back in a minute. About five minutes afterwards a nurse came to him and told him the boy was in a fit, and he was at once seen again. It was now evident that serious injury had resulted from the accident, and the parents were told that it would be best for them to let the boy come into the hospital. To this they strongly objected, and several minutes were spent in convincing them that this was a necessary course to pursue. Consent having at length been obtained, he was at once transferred to the ward, and seen immediately by Mr. Turner, the house surgeon. In ten minutes' time Mr. Dunn, an assistant surgeon to the hospital, saw him, and it was decided that no operation was practicable, as a diagnosis of meningitis was made. The boy improved a little during the day, but died suddenly next morning. At the post-mortem examination the diagnosis of meningitis was found to be correct, while the track of the stick through the orbit was traceable with great difficulty. At the inquest Mr. FitzHugh is reported to have said that Mr. Stoehr was away on his holidays; this is incorrect. What he did say was that Mr. Booker, on whose responsibility he was sent from the hospital on the Friday, was away on his holidays. In conclusion, neither Mr. Stoehr nor Mr. FitzHugh were subpœnaed to attend the inquest; the latter went hurriedly at the request of Mr. Turner without knowing even for what case he had been sent."

### DRUNK OR DYING.—THE CASE OF JAMES FISHER.

Last week we mentioned in "Notes and News" another instance of the fatal confusion which occurs between drunk and dying in certain cases applying for treatment at the hospitals. The patient, James Fisher, a carman, who fell from his van at Islington, was taken to Guy's Hospital, whence he was moved to the police station as drunk. He was finally sent from the police station to the Rotherhithe Infirmary, where he died from the effects of a fracture of the skull. We have received the following communication from the authorities at Guy's Hospital on the subject:—

"On Wednesday night the carman Fisher was brought into the front surgery by the police on an ambulance with a history of his having fallen from a van. He was seen by the house surgeon a few minutes after his arrival. The injuries from which he was suffering were a slight scalp wound about one inch long, scarcely more than an abrasion, the skin not being completely divided. This was situated about three inches above the left ear. There was a lacerated wound of the left ear about three-quarters of an inch in length, situated vertically above the meatus. The ear was cleaned and no blood was found to be coming from the meatus. There was no epistaxis. He was not unconscious, and only very slightly drowsy. He was able to walk, though unsteadily, and was able to speak and answer questions. There was a strong odour of alcohol about his breath, and he admitted being 'three parts drunk.' The pupils were moderately dilated, equal, and both reacted, and the pulse normal. He was detained for one and a half hours, and his condition remaining unchanged, was sent away in charge of the police. On his arrival at the police-station he made no reply when charged with being drunk, though according to the police evidence, he was able to speak rationally and walk about. He remained in the same condition the whole night, being able to speak and walk about, and made no complaint. Next morning he asked for breakfast. At about eight a.m. the first bad symptom was noticed—he complained of feeling dizzy, and soon afterwards had a right-sided convulsion. The divisional surgeon then saw him, and stated at the inquest that the man then smelt strongly of alcohol. He was at once removed to the infirmary, where he died in about two hours. At the post-mortem a fracture was found involving the greater wing of the sphenoid running to the body of the sphenoid, and having a spur directed backwards running across the anterior part of the petrous bone. The brain was lacerated, and there was a good deal of blood-clot which was entirely subdural. The stomach contained at least one and a half pints of foul-smelling fluid and no solid, and there was chronic gastritis. The liver was enlarged and fatty. There were two fractured ribs—the third and fourth on the left side—far back beneath the scapular muscles, where a diagnosis was admittedly practically impossible."

### ROYAL ARMY MEDICAL CORPS.

The following is a list of successful candidates in the Royal Army Medical Corps at the recent examination in London: F. Warren, 2,638 marks; J. E. Hodgson, 2,617; H. P. W. Barrow, 2,602; M. H. G. Fell, 2,511; T. C. Lauder, 2,415; J. G. Gill, 2,292; W. B. Winkfield, 2,266; G. H. Goddard, 2,211; G. W. G. Jones, 2,183; J. W. H. Houghton, 2,117; H. S. Taylor, 2,101; A. L. Scott, 1,991; J. W. Leake, 1,969; R. H. Lloyd, 1,935; D. E. Curme, 1,892; G. M. Goldsmith, 1,848.

